# New Players in Immunity to Tuberculosis: The Host Microbiome, Lung Epithelium, and Innate Immune Cells

**DOI:** 10.3389/fimmu.2018.00709

**Published:** 2018-04-10

**Authors:** Nancy Gupta, Rakesh Kumar, Babita Agrawal

**Affiliations:** ^1^Department of Laboratory Medicine and Pathology, Faculty of Medicine and Dentistry, University of Alberta, Edmonton, AB, Canada; ^2^Department of Surgery, Faculty of Medicine and Dentistry, University of Alberta, Edmonton, AB, Canada

**Keywords:** tuberculosis, microbiota/microbiome, innate lymphoid cells, alveolar immune system, mucosa-associated invariant T

## Abstract

Tuberculosis (TB) is a highly contagious infection and devastating chronic disease, causing 10.4 million new infections and 1.8 million deaths every year globally. Efforts to control and eradicate TB are hampered by the rapid emergence of drug resistance and limited efficacy of the only available vaccine, BCG. Immunological events in the airways and lungs are of major importance in determining whether exposure to *Mycobacterium tuberculosis* (*Mtb*) results in successful infection or protective immunity. Several studies have demonstrated that the host microbiota is in constant contact with the immune system, and thus continually directs the nature of immune responses occurring during new infections. However, little is known about its role in the eventual outcome of the mycobacterial infection. In this review, we highlight the changes in microbial composition in the respiratory tract and gut that have been linked to the alteration of immune responses, and to the risk, prevention, and treatment of TB. In addition, we summarize our current understanding of alveolar epithelial cells and the innate immune system, and their interaction with *Mtb* during early infection. Extensive studies are warranted to fully understand the all-inclusive role of the lung microbiota, its interaction with epithelium and innate immune responses and resulting adaptive immune responses, and in the pathogenesis and/or protection from *Mtb* infection. Novel interventions aimed at influencing the microbiota, the alveolar immune system and innate immunity will shape future strategies of prevention and treatment for TB.

## Introduction

Tuberculosis (TB), caused by *Mycobacterium tuberculosis* (*Mtb*), is responsible for over one billion deaths in the last 200 years, more than any other single pathogen. Despite the increased global attention, the expansion of therapeutic drug regimens, and the widespread use of existing vaccine, ~1.8 million people still die every year as a result of this devastating disease (1). Furthermore, increasing outbreaks of drug-resistant TB and TB/HIV co-infection pose a significant threat to treating and preventing further transmission (2). An epidemiological model has estimated that without adequate treatment and prophylactic measures, TB will infect ~225 million and kill 79 million people between the years 1998 and 2030 (3).

Available anti-TB drugs have a profound effect on drug-susceptible TB with >90% cure rates. But resistance to almost all of the available drugs is rapidly emerging in the form of multi-, extremely- and totally drug-resistant TB (MDR, XDR, and TDR-TB), and the development of new anti-TB drugs severely lags behind (4). The current vaccine, bacille Calmette–Guérin (BCG), has been available for ~70 years, but it is not very effective and provides only partial and inconsistent protection (0–70%) (5). Furthermore, the incidence of concurrent infection with different strains of *Mtb* and exogenous re-infection following *Mtb* drug-treatment suggest that adaptive immunity to *Mtb* is not solely protective. Extensive clinical and animal studies have examined an essential role of adaptive immunity in controlling mycobacterial growth or replication. In addition, in the last 20 years, a concerted worldwide effort has been prompted to develop a new preventive and/or therapeutic TB vaccine. Unfortunately, none of them showed sufficient efficacy through clinical trials. Clearly, something is missing. Development of a new effective vaccine against TB remains challenging due to a poor understanding of immune-correlates of protection and disease pathogenesis (6).

**Figure 1:**
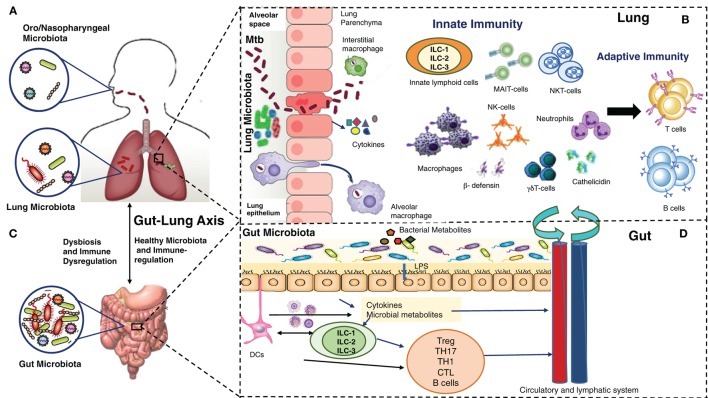
Multiple players in *Mycobacterium tuberculosis* (*Mtb*) infection and immunity: lung microbiota, lung epithelium, gut–lung axis, gut microbiota, innate and adaptive lymphocytes. **(A)** The upper (oro/nasopharyngeal) and lower respiratory (lung) microbiota. **(B)** Alveolar epithelial cells secrete cytokines, opsonins, and antimicrobial peptides upon mycobacterial infection. Alveolar macrophages/interstitial macrophages constitute the first line of immune defense and also the first port of entry during mycobacterial infection, but their interaction with lung microbiota is not yet known. Innate lymphocytes, such as MAIT, NK, NKT, γδ T cells, and innate lymphoid cells (ILCs) become activated, and their coordination leads to subsequent expansion/modulation of adaptive T and B cells. Dendritic cells transport *Mtb* antigens to draining lymph nodes to promote *Mtb*-specific immunity. **(C)**. In a healthy state, the gut microbiota regulates lung immunity and influences the lung microbiota. Dysbiosis caused by anti-TB therapy in the gut can lead to dysregulation of immune responses in the lung. **(D)**. The intestinal microbes and their metabolites regulate ILCs directly, or through cytokines produced by gut epithelium or DCs. ILCs and DCs in turn regulate adaptive T and B cells in the gut which migrate systemically and to lungs. Also, a combination of signals from microbes leads to migration of DCs to the draining lymph nodes, where DCs promote activation of various T cell subsets and B cells. During mycobacterial infection, cells activated in gut-associated lymphoid tissue (GALT) and mesenteric lymph nodes migrate to the lungs where they promote protective immunity and influence the lung microbiota. Bacterial metabolites are also directly transported to the lungs to influence the lung immunity against mycobacterial infection. The exact pathways of interactions between the components of **(A–D)** are still being explored.

Consequently, novel therapeutic strategies, which could control ongoing infection and associated pathogenesis, reduce or prevent recurrence, and effectively deal with increasing drug resistance, are needed to control the global epidemic of TB. A positive aspect to this is that 90% of immunocompetent individuals exposed to *Mtb* do not develop active disease, clearly suggesting a critical role of host immunity to prevent and/or clear the early infection. Most research targeting host immunity has so far focused on generating and maintaining antigen-specific adaptive immune responses against *Mtb* as an effective way to prevent and/or treat *Mtb* infections. Despite significant effort and resources, they have not been very successful yet. There is a serious need to more comprehensively understand the network of immunological mechanisms underlying protection and/or clearance of TB infection, which allow a precise balance between host protective responses and immune pathogenesis. This extensive undertaking must take into account relatively newly identified but dominant players in host immunity: the microbiome/microbiota, the epithelium and the innate immune cells, in addition to adaptive immunity (Figure 1). In this review, we provide a brief overview of our current understanding of the gut–lung microbiota, the airway epithelium, innate immune cells, and their collective interaction with *Mtb*.

## Microbiota and TB

The microbiota have emerged as a new biomarker of human health as it plays a vital role in maintaining normal health, developing and educating the immune system, and providing protection against pathogens. There is an elegant mutualistic interaction between the human host and microbiota. Recent studies have revealed that changes in the microbiota of healthy individuals parallel various pathological conditions (7–12). How to apply this knowledge to improve human health is at the very least “convoluted.” Still, it is an important beginning that needs a dedicated effort to succeed.

Early studies of human microbiota fundamentally focused on the roles of intestinal commensals and their metabolites in regulating various inflammatory and metabolic disorders. It is now becoming apparent that the immunological function of gut microbiota extends far beyond the local environment of the GI tract, immune homeostasis, and immune defense against enteric bacterial and viral infections (13–15). Evidence is mounting in support of a dominant and decisive role of gut as well as lung microbiota in shaping and modulating immune responses in the prevention, pathogenesis, and treatment of respiratory diseases (16–21). Alteration in gut microbiota, resulting in immunological dysregulation, is associated with the development of chronic respiratory diseases, such as allergy, asthma, COPD, and cystic fibrosis (21–28).

The role of microbiota during mycobacterial infection remains largely unexplored. To date, only a few studies have focused on studying the changes in the gut and/or lung microbiota during *Mtb* infection and the risk of progressive TB. The cross-talk between the lung and gut microbiome, as well as innate and adaptive immune cells that may link these two mucosal sites, appear to be important in the prevention, pathogenesis, and treatment of TB.

There are several studies that point toward a prominent role of gut microbiota in stimulating and fostering the development and maintenance of immune responses during *Mtb* infection.

*Helicobacter pylori* are commensal bacteria inhabiting the stomach of ~50% of the world’s population. Usually *H. pylori* are harmless gut habitants, but in ~10% of the people harboring them, they can lead to gastritis, peptic ulcer, and gastric cancer. The acidic gastric environment has long been considered to be a sterile environment, but now it is well recognized that stomach also has a distinct microbial community, albeit with much lower bacterial density than intestine and colon. Interestingly, *H. pylori* are uniquely adapted to colonize in the human stomach, by generating ammonia and ${\text{HCO}}_{3}^{-}$, which can neutralize the gastric acid. The infection with *H. pylori* results in the induction of inflammatory responses, which are unable to clear the infection but which drive the chronic gastric immunopathology. Long-term colonization and neutralization of gastric acid by *H. pylori* may also contribute to the alteration in the microbiota leading to dysbiosis (29). Perry et al. have reported that *H. pylori* seropositive individuals with latent TB had high TB antigen-specific Th1 responses and IFN-γ production and were less likely to develop active TB disease, compared to *H. pylori* seronegative individuals (30). Furthermore, it has been shown in a mouse model that alteration of gut microbiota in early life dominated by the bacterium *Helicobacter hepaticus* intensely influences the magnitude and protective efficacy of immune responses to subunit vaccine Ad85A (31). In addition, stool microbiota rich in *Bifidobacterium* spp. was associated with increased PPD-specific T cell responses after BCG vaccination in infants. This study suggested a role of the neonatal gut microbiome in modulating vaccine-induced immunity and the effectiveness of the BCG vaccination right after birth (32).

Antibiotic-induced alteration in gut bacterial composition before and after *Mtb* challenge has been shown to promote higher susceptibility to *Mtb* infection and dissemination of mycobacteria in liver and spleen (33). Disruption of gut microbiota has also been demonstrated to modulate adaptive immune responses to TB, with increased numbers of regulatory T cells and reduced frequency of IFN-γ and TNF-α-secreting CD4^+^ T cells upon *Mtb* challenge. Interestingly, fecal transplantation was shown to reconstitute the gut microbiota, restore anti-TB immunity, and prevent dissemination of TB to other organs (33). Recently, a meta-analysis of published studies has shown that narrow-spectrum first line anti-TB drugs have dramatic effects on microbiome diversity and immunity, which persists even after the completion of TB drug therapy. Further, the persistent dysbiosis that accompanies curative anti-TB treatment could contribute to post-treatment susceptibility to reinfection, not only mycobacterial but also with other diseases linked with altered immune responses (34, 35).

The studies described above clearly establish the role of gut microbiota on the quality of TB immunity in the lungs. Although previously unrecognized and unappreciated, the respiratory tract also harbors a rich microbiota, albeit at smaller levels than gut. Interestingly, there is a continuum of bidirectional cross-talk between gut and lungs through the so-called gut–lung axis, mediated by passage of bacteria, bacterial products, and inflammatory mediators through blood and lymphatics and directly through aspiration. The lung microbiota is more dynamic and transient than in the gut as the lung is a low bacterial burden organ and is continually influenced by microbial immigration and elimination (36–38). In the upper respiratory tract (URT), nasal and oral cavities contain distinct microbiota: the nasal cavity is enriched with *Streptococcus, Acinetobacter, Lactococcus, Staphylococcus*, and *Corynebacterium* whereas *Prevotella, Streptococcus, Fusobacterium, Neisseria, Leptotrichia*, and *Veillonella* dominate in the oral cavity (39). Furthermore, the microbiota in the URT is constantly exposed to airborne and ingested diet-associated microbes.

The limitation in obtaining lower respiratory tract (LRT) samples remains a major impediment in studying the composition of the lung microbiome in health and disease. Previously, the LRT or lungs were considered a sterile environment. But a number of investigations have conclusively demonstrated that a healthy LRT also has abundant microbiota similar to the predominant phyla detected in the healthy intestine: *Firmicutes, Bacteroidetes*, and *Proteobacteria*. However, the LRT has 100- to 10,000-fold fewer bacteria than the URT (40).

The role of the lung microbiota is beginning to be appreciated, although its contribution to pulmonary diseases still remains unclear. The lung microbiota in healthy humans more closely resembles that of the oropharynx than of the nasopharynx (41). Emerging evidence suggests that microbiota residing in lungs are crucial to immune fitness, and provide essential signals for the development and appropriate function of the immune system and resistance to inflammatory and infectious disease. Despite many advances, our understanding of the changes in the composition of the microbial communities in the lungs in the context of TB is only starting to emerge.

The data collected so far suggest the lung microbiome changes in disease pathogenesis, treatment failure and recurrent TB infection, however, several contradictory findings have been reported in the characterization of the microbial diversity associated with TB disease.

Cui et al. (42) reported that bacterial diversity was significantly higher among sputum isolates of TB patients than of healthy controls. In addition, healthy participants demonstrated a strong clustering pattern (235/614 total genera) while pulmonary TB patients had a more scattered pattern (564/614 total genera). Furthermore, many foreign bacteria, such as *Stenotrophomonas, Cupriavidus, Pseudomonas, Thermus, Sphingomonas, Methylobacterium, Diaphorobacter, Comamonas, Mobilicoccus*, and so on, were unique to, and widely distributed among, the pulmonary TB patients (42). By contrast, Cheung et al. (43) reported that there was no difference in microbial diversity among TB patients and healthy controls and no direct correlation between microbial diversity and TB disease. This study, however, had a small sample size and comparisons were made between sputums from TB patients and respiratory secretions from healthy controls (44). In a more recent study, Krishna et al. (45) reported that *Firmicutes* and *Actinobacteria* dominate the sputum of TB patients, while *Bacteroides* and Proteobacteria were significantly higher in sputum samples of healthy controls. Similar to Cui et al., they reported the presence of opportunistic bacteria in sputums of TB patients prior to anti-TB therapy (45). In another study of a relatively large cohort (total 95) of new TB patients, recurrent TB and treatment failure TB patients, sputum analysis suggested that the presence of foreign bacteria and changes in lung microbiome are not only associated with the onset of disease but also with the recurrence and failure of anti-TB therapy (44).

In addition to microbiota, microbial metabolic activity and their products may also influence the outcome of TB infection. Increased production of short-chain fatty acids such as butyric and propionic acids by anaerobic *Prevotella* in the LRT of HIV-infected individuals are positively correlated with increased incidence of active TB (46). Butyrate inhibits mycobacterial antigen-specific IL-17 and IFN-γ responses and causes an increase in *Mtb* antigen-specific FOXP3^+^ regulatory T cells in the lungs, suggesting an active role of microbial metabolites in immunity to *Mtb* (47, 48).

Recent studies of the human microbiome have mainly been focused on the role of bacteria (bacteriome) and their components. But new evidence suggests that non-bacterial microbiota residing in gut and lungs, fungi (mycobiome) and viruses (virome), could be critical in modulating immune responses, and disease and treatment outcomes (49–51). Yet these relationships remain largely unexplored. In TB patients, two genera of fungus, *Candida* and *Aspergillus*, were found in abundance in both sputum and oropharyngeal samples (52).

## The Lung Epithelium

The respiratory tract epithelium serves as the first protective barrier in defense against respiratory/mucosal pathogens. Non-hematopoietic airway epithelial cells (AECs) are now emerging to play a critical, active role in interacting with the microbiota, initiating and expanding local innate immune responses and subsequent adaptive immunity, thereby preventing pathogens from invading lung parenchyma and remodeling tissue after a pathogenic or inflammatory damage (53–56). AECs express a number of pattern-recognition receptors (PRRs), which bind to pathogen-associated molecular patterns of various pathogens. Upon sensing pathogens, AECs secrete antimicrobial effector molecules, peptides, enzymes, reactive nitrogen and oxygen species and a range of cytokines, chemokines, and growth factors, which help in the recruitment and communication with immune cells and contribute to the initiation of innate immune responses critical for early control of an infection (57–61). The current knowledge of the contribution of AECs in the induction of innate immune responses and their possible role in pathogenesis or protection in context of mycobacterial infection is described in the following section.

Airway epithelial cells are the very first host cells encountering *Mtb* bacilli after aerosol inhalation and play the most prominent role in the binding, recognition, and internalization of mycobacteria followed by initiation of an immune response (62). They express a variety of PRRs, such as TLRs, RIG-1-like receptors, NOD-like receptors, and C-type lectins, as well as surfactant proteins that bind to the components of the mycobacterial cell wall (63–66). Epithelial recognition of *Mtb* activates several signaling pathways and induces production of cytokines (TNF-α, IFN-γ, GM-CSF, IL-6, IL-10, etc.) and chemokines (IL-8, IP-10, IL-27, MCP-1, MIG) (67–71). Early secretion of these soluble immune mediators allows communication between these immune AECs and other immune cells to subsequently initiate recruitment and activation of monocytes, phagocytes, lymphocytes, and polymorphonuclear leukocytes to the lungs (72). Recently, a study using human primary bronchial epithelial cells *in vitro* revealed that epithelium was inert to direct *Mtb* infection but was a potent responder to cytokines (IL-1β and type I interferons) released by infected macrophages, allowing an efficient cross-talk (73). Stimulation of AECs by BCG also leads to early activation of neutrophils that positively affect the protective efficacy against pulmonary TB through the induction of Th1 and Th17 cells (74). Interestingly, AECs express MHC I molecules and can directly present intracellular antigens to resident CD8^+^ T cells. It has been shown that *Mtb* are localized in the late endosomal vacuole of lung epithelial cells, and their antigens are efficiently presented to CD8^+^ T cells to stimulate IFN-γ production (75). Thus, AECs may play a critical role in initiating protective adaptive immunity to mycobacterial infections.

In response to mycobacterial infections, AECs were shown to produce antimicrobial peptides and nitric oxide (NO) in several *in vitro* and *in vivo* studies. AECs secrete antimicrobial peptides cathelicidin (LL-37), β-defensin-2, and hepcidin that have been shown to play a critical role in innate immunity against mycobacteria (76–78). Human alveolar epithelial cell line A549 has also been shown to produce LL-37, hepcidin, and NO upon stimulation with BCG. Human epithelial cells have also been shown to produce β-defensin-2 upon exposure to BCG, which enhances host defense to control *Mtb* infection (79–81). Airway epithelium, therefore, plays a non-redundant role in initiating and shaping the innate immune response at the very first site of exposure and influences the outcome of *Mtb* infection (73, 82). In addition, through TGF-β production, alveolar epithelial cells also play an important immunoregulatory role in maintaining epithelial integrity and preventing immune-mediated destruction by limiting inflammation (83). Recent studies have also revealed a comprehensive interaction of airway epithelium with lung microbiota. How these interactions are affected during *Mtb* infection, and also whether or how they play a significant role in active TB, latency and reactivation from latency, needs to be investigated.

## Innate Immunity

The early innate immune interactions between mycobacteria and the host are crucial and predictive of the eventual outcome of infection as well as maintenance of long-term memory responses. And yet they are poorly understood due to a historic emphasis on adaptive immunity as a major player in TB immunity. It is now being recognized that early immune events after exposure to *Mtb* are not “silent” in humans but are rather robust, and characterized by inflammatory processes and thoracic lymph node involvement, regardless of infection trajectory. Notably, these initial events are successful in restraining the infection to a large extent since most infections do not progress to active TB disease.

Several case contact studies have confirmed that exposure to *Mtb* does not always lead to TB infection. In high TB epidemic areas, half of the exposed people never get infected with *Mtb* and remain negative to the tuberculin skin test (TST) and IFN-γ release test, while half become TST positive with the absence of Th1-type adaptive immunity against *Mtb* antigens. In these settings, it is highly likely that *Mtb* was inhaled, contained and cleared before the development of adaptive immunity. In this regard, quantitative assessment of innate immune responses in whole-cell *Mtb* stimulation assays revealed an unexpected cytokine signature: TST-negative individuals demonstrated lower TNF-α induction in response to LPS stimulation compared to TST-positive people. These results clearly demonstrated that measurement of a single parameter such as TNF-α is not sufficient and there is a need for deeper understanding of the roles played by various innate immune functionalities (84–87). Activation of TLR2 on human and mouse macrophages by microbial lipoproteins has been shown to kill intracellular *Mtb*, providing direct evidence of innate immune-mediated clearance of *Mtb* (88). These studies suggested that innate immune responses are associated with the early clearance of *Mtb* before the onset of adaptive immunity. Furthermore, it is conceivable that examining gut–lung microbiota and early immune events in the subjects with resistance to and/or self-clearing infection with *Mtb* would provide essential information regarding protective immunity. Growing evidence suggests that the innate immune system can also produce pathogen-specific responses and mount resistance to secondary infections through “innate immune memory” or “trained immunity” (89–91). Successful treatment for mycobacterial infection requires complete clearance of mycobacteria, resolution of infection-induced inflammation and repair/remodeling of lung epithelium. Multiple studies have examined the role of various innate immune cells recruited to lungs following *Mtb* exposure/infection, e.g., neutrophils, NK cells, NKT cells, and γδ T cells (92–97). However, the role of mucosal (lung)-resident innate lymphocytes in *Mtb* pathogenesis and clearance remains to be established. We will briefly discuss the possible role of some of these potential new players in protective immunity against TB in the following section.

Neutrophils are among the first immune cells that migrate to the infection site during *Mtb* infection and play a crucial role in the development of innate and acute inflammatory responses (98). During *Mtb* infection, neutrophils produce and secrete antimicrobial enzymes (α-defensins, matrix metalloproteases, lactoferin, and lipocalin) to restrict the growth of mycobacteria within macrophages, and promote apoptosis of infected macrophages, thereby limiting *Mtb* survival within the host. Upon stimulation with *Mtb*, they also secrete chemokines (IP-10, MCP-1, MIP-1α/β) and pro-inflammatory cytokines (IFN-γ and TNF-α) to recruit and activate other immune cells (99, 100). However, these effector molecules also mediate lung tissue damage and a sustained, hyper-activated inflammatory response. Neutrophils are the second most abundant cells, after lymphocytes, found in bronchoalveolar lavage (BAL) and sputum samples of active pulmonary TB patients (101, 102). Furthermore, neutrophils have been reported to highly express programmed death ligand-1 (PDL-1) and type I IFN-inducible genes in the blood of active TB patients (103, 104). It is still controversial whether the increased expression of PDL-1 on neutrophils is associated with suppression of protective immunity or with the resolution of inflammation.

NK cells are prominent cellular components of innate immunity that play a central role in clearing the intracellular pathogens. NK cells mediate their function through cellular cytotoxicity and production of a range of cytokines (105, 106). In acute mycobacterial infection, NK cells have been shown to possess increased cytotoxic activity, IFN-γ and TNF-α production, and upregulation of activation marker NKG2D/NKp46 (107–109). They have been also shown to lyse infected monocytes, alveolar macrophages, and *Mtb*-expanded T regulatory cells, induce γδ T cell proliferation, and promote IFN-γ production from CD8^+^ T cells (110, 111). It has been shown that depletion of NK cells in mice at the time of BCG vaccination enhances the expansion of T regulatory cells and impedes the vaccine-induced protective immunity against challenge with Mtb H37Rv (112). In another study, vaccination of mice with BCG was shown to expand memory-like NK cells in an antigen-dependent manner, which was suggested to provide protection against subsequent *Mtb* infection (113). Expansion of IL-21-dependent memory-like NK cells was also seen in people with latent TB (114). By contrast, in patients with active TB, NK cells have been reported with reduced cytotoxicity, depressed IFN-γ production, and lowered expression of NKp30- and NKp46-activating receptors (115, 116). In orchestrate acute inflammation highlighted the importance of NK cells during mycobacterial infection, especially in TB-HIV co-infected patients (93). A full understanding of the role of NK cells in antimycobacterial immunity may open new possibilities for the development of immunotherapeutic strategies against TB.

NKT cells are innate immune cells expressing both NK and T cell markers and possess effector as well as regulatory functions. NKT cells are classified as type 1 or invariant NKT (iNKT) with restricted TCRs and type II or heterogeneous NKT with less restricted TCRs (117–120). Growing evidence suggests that NKT cells mediate protection against *Mtb* in both humans and mouse models (121). In mice, administration of α-GalCer (a known iNKT agonist), both alone and in combination with anti-TB drugs, improved the outcome of *Mtb* infection (122). Incorporation of α-GalCer in BCG vaccine has been shown to enhance the induced immune responses (123). Also, patients with active TB were found to have dysfunctional NKT cells with increased expression of inhibitory molecule PD-1 (94). Recently, it has also been shown that NKT cells isolated from pleural fluid of TB patients produce IFN-γ, TNF-α, IL17, IL-2, and IL-21 upon *ex vivo* stimulation with antigen (124, 125). It has been suggested that NKT cells become activated during the early infection with pulmonary TB, and actively participate to resolve *Mtb* infection (126). Whether, and to what extent NKT cells are associated with early innate resistance to mycobacterial infection is not clear yet.

γδ T cells are a distinct subset of CD3^+^ T cells, which carry a T cell receptor encoded by Vγ and Vδ gene segments. They recognize unprocessed, non-peptide phosphate antigens in a non-MHC restricted manner (127). γδ T cells represent an early defense against pulmonary TB and serve as a link between innate and adaptive immunity. During the initial phase of *Mtb* infection, γδ T cells are recruited in the lungs, which express IFN-γ and IL-17 along with cytotoxic effector function (128). Increased frequency of γδ T cells has been shown in lungs in patients with active TB (129). Studies in both mice and humans suggest that γδ T cells, expanded after BCG vaccination, are capable of restricting mycobacterial growth in a perforin- and granulysin-dependent manner (130). γδ T cells also elicit protective immune responses through their interaction with NKs, DCs, and CD8^+^ T cells (131). Further studies are needed to define a precise role of γδ T cells in protective immunity to *Mtb* infection.

Mucosa-associated invariant T (MAIT) cells are prevalent in blood and mucosal sites in humans. They are unique innate cytotoxic T cells that emerge from the thymus as effectors, and thus act as immediate effectors in response to pathogens. MAIT cells have a limited T cell receptor repertoire, act in a non-classical MR1-restricted manner, get stimulated by vitamin B metabolites common in bacteria and yeast, and respond to host cells infected with bacterial pathogens with cytokine production and cytotoxicity, without prior priming and stimulation (132). MAIT cells have been shown to produce IFN-γ and TNF-α in response to *Mtb*-infected cells and also induce target cell lysis through secretion of cytotoxic granules (133). Mice lacking MR1 or MAIT cells were shown to have increased mycobacterial loads upon aerosol challenge with *M. bovis* (134). In patients with active TB, the frequency of MAIT cells was decreased in peripheral blood but increased in the lungs compared to healthy individuals, suggesting that MAIT cells migrate from the periphery to the mucosal site of infection to provide protection during infection (135). Also, MAIT cells from the peripheral blood of active TB patients exhibited impaired cytokine (IFN-γ, TNF-α, IL-17) and cytotoxic response (granulysin and granzyme B) upon stimulation with *Mtb* antigens. Furthermore, MAIT cells from peripheral blood of active TB patients had elevated expression of programmed death-1 (PD-1) molecules and blockade of PD-1 resulted in enhancement in antigen-stimulated IFN-γ production (136). Early clearance of mycobacteria after exposure has been associated with MAIT memory cells that have previously encountered non-tuberculous environmental mycobacteria (137). Therefore, MAIT cells appear to be an important component of innate immunity against TB, which need further exploration.

In recent years, innate lymphoid cells (ILCs) have emerged as a new family of innate counterparts of T helper lymphocytes. ILCs are derived from an Id2-dependent lymphoid cell progenitor cell population abundant at mucosal surfaces, and play a significant role as a first line of defense against pathogens as well as in immune homeostasis. ILCs rapidly respond to microbial and cytokine signals and are potent innate cellular sources of multiple pro-inflammatory and immunoregulatory cytokines (138). ILCs have also been shown to play a critical role in modulating adaptive immunity toward tolerance and/or protective immunity (139). There is considerable phenotypic and functional heterogeneity in the mature ILC family, and broadly three groups of ILCs (ILC1, ILC2, and ILC3) have been defined based on shared expression of surface markers, transcription factors, and effector cytokines (140). ILCs orchestrate acute inflammation to promote immunity to infection as well as promote the resolution of infection-mediated inflammation and damage of tissues in lungs and intestine (141). They have also been shown to promote the barrier function of lung epithelium and lung tissue homeostasis in multiple chronic infectious and inflammatory diseases of the respiratory tract. Lung tissue destruction and/or remodeling is a key process in the development of TB disease. However, the role of ILCs in pathogenesis and/or clearance of mycobacterial infection remains largely unknown.

A significant reduction in all ILC populations was reported among 44 subjects diagnosed with active drug-susceptible and drug-resistant TB infections, in comparison to healthy controls (*p* < 0.0001) (142). Treatment of drug-susceptible TB was reported to restore the levels of ILC1 and ILC3 but not ILC2. Furthermore, ILC populations isolated from lungs of TB-infected individuals expressed high levels of activation markers CD69, CD25, and CCR6 compared to NK and T cells (142). This study suggests that modulation of the ILC population during *Mtb* infection might possibly have a significant pathogenic and/or protective role in TB disease.

Innate lymphoid cells also interact with the microbiota and the mucosal epithelium in a major way that induces tolerance or active adaptive immunity, and thus could shape the success or failure of a pathogen such as *Mtb* in establishing an active long-term infection. However, our understanding of the details underlying the pivotal role of microbiota–epithelium–ILCs–T cell interactions during *Mtb* infection still remains scarce. Such knowledge would be critical in understanding protective immunity against *Mtb* and developing effective and selective host-directed therapeutics.

## Conclusion and Future Directions

Our understanding of microbiota and their widespread role in infectious, inflammatory, metabolic diseases, and homeostasis has expanded in recent years, but numerous challenges and unanswered questions remain. Detailed analyses of the role of microbiota in active TB disease, latency, reactivation from latency and clearance with or without antibiotic treatment remains to be thoroughly investigated. In addition, instead of examining microbiota in TB disease on its own, a more in-depth understanding of their interactions with airway epithelium and the innate and adaptive immune systems will be required. Such studies will help translate these intricate interactions to support the development of new therapeutic interventions that target the dynamic association among these processes.

## Author Contributions

NG, BA, and RK contributed equally to the writing of this review article. RK and BA are co-corresponding authors.

## Conflict of Interest Statement

The authors declare that the research was conducted in the absence of any commercial or financial relationships that could be construed as a potential conflict of interest.
